# Adsorption Removal of Environmental Hormones of Dimethyl Phthalate Using Novel Magnetic Adsorbent

**DOI:** 10.1155/2015/903706

**Published:** 2015-07-16

**Authors:** Chia-Chi Chang, Jyi-Yeong Tseng, Dar-Ren Ji, Chun-Yu Chiu, De-Sheng Lu, Ching-Yuan Chang, Min-Hao Yuan, Chiung-Fen Chang, Chyow-San Chiou, Yi-Hung Chen, Je-Lueng Shie

**Affiliations:** ^1^Graduate Institute of Environmental Engineering, National Taiwan University, Taipei 106, Taiwan; ^2^Department of Cosmetic Science and Application, Lan-Yang Institute of Technology, Yilan 261, Taiwan; ^3^Department of Chemical Engineering, National Taiwan University, Taipei 106, Taiwan; ^4^Department of Chemical Engineering and Biotechnology, National Taipei University of Technology, Taipei 106, Taiwan; ^5^Department of Environmental Science and Engineering, Tunghai University, Taichung 407, Taiwan; ^6^Department of Environmental Engineering, National I-Lan University, Yilan 260, Taiwan

## Abstract

Magnetic polyvinyl alcohol adsorbent M-PVAL was employed to remove and concentrate dimethyl phthalate DMP. The M-PVAL was prepared after sequential syntheses of magnetic Fe_3_O_4_ (M) and polyvinyl acetate (M-PVAC). The saturated magnetizations of M, M-PVAC, and M-PVAL are 57.2, 26.0, and 43.2 emu g^−1^ with superparamagnetism, respectively. The average size of M-PVAL by number is 0.75 *μ*m in micro size. Adsorption experiments include three cases: (1) adjustment of initial pH (pH_0_) of solution to 5, (2) no adjustment of pH_0_ with value in 6.04–6.64, and (3) adjusted pH_0_ = 7. The corresponding saturated amounts of adsorption of unimolecular layer of Langmuir isotherm are 4.01, 5.21, and 4.22 mg g^−1^, respectively. Values of heterogeneity factor of Freundlich isotherm are 2.59, 2.19, and 2.59 which are greater than 1, revealing the favorable adsorption of DMP/M-PVAL system. Values of adsorption activation energy per mole of Dubinin-Radushkevich isotherm are, respectively, of low values of 7.04, 6.48, and 7.19 kJ mol^−1^, indicating the natural occurring of the adsorption process studied. The tiny size of adsorbent makes the adsorption take place easily while its superparamagnetism is beneficial for the separation and recovery of micro adsorbent from liquid by applying magnetic field after completion of adsorption.

## 1. Introduction

Emerging contaminants (ECs), such as hormones, have spread in various environmental media due to wide use of materials or products containing these substances or compounds. Most of these contaminants were discharged into aquatic environments [1–16]. The ECs were also found in sediments [1, 6, 8, 17, 18], soil [8, 9, 19], air [20, 21], waste [19], and biota [1, 22, 23]. There has been a great ongoing concern, even at trace levels, on their potential, adverse effects on human health and ecological systems affecting biological and cell mechanisms [1, 3, 9, 11–13, 15, 24]. Among the environmental hormones encountered, phthalate acid esters or phthalate esters (PAEs) have drawn much attention [18, 20, 21, 25–30].

PAEs are derivatives of phthalate acid. They have been applied for the manufacturing of polyvinyl chloride (PVC), polypropylene (PP), polyethylene (PE), and polystyrene (PS) and used as plasticizer, binder, and coating and printing ink [21, 27]. Due to their endocrine disruption effects, many countries have either regulated the upper limiting concentration or restricted the use of PAEs in plastic toys. The contaminated sources include plastics factories, industrial and domestic wastewater treatment plants, landfills, reviver sediment, and sewage sludge [18, 20, 25, 27–30]. The solubility of PAEs in water is low. Among the commonly used PAEs, dimethyl phthalate (DMP) has the solubility in water of 4000 mg L^−1^ or 0.4% [31], which is comparatively higher than most of other PAEs such as di-ethyl phthalate (DEP) (1080 mg L^−1^), di-hexyl phthalate (DHP) (slightly soluble with 0.05% or 500 mg L^−1^ max), di-n-butyl phthalate (DBP) (13 mg L^−1^), benzyl butyl phthalate (BBP) (2.69 mg L^−1^), di-(2-ethyl hexyl) phthalate (DEHP) (0.3 mg L^−1^), di-iso-nonyl phthalate (DINP) (<0.01 mg L^−1^), di-cyclohexyl phthalate (DCHP) (negligible solubility), di-n-octyl phthalate (DNOP) (water insoluble), and di-iso-decyl phthalate (DIDP) (water insoluble). Thus, DMP was chosen as a model compound for the remediation of contaminated aquatic water and the treatment of waste water.

The natural attenuation mechanisms which include chemical breakdown, biodegradation, and photolysis can decompose the PAEs [25]. The halftimes vary from days to years, depending on the environment especially the temperature and the properties of PAEs such as structure and length of functional groups. However, it takes a long time. Thus, many techniques have been used to treat the PAEs-containing water and waste, including physical treatments of adsorption [32, 33] and membrane processes such as ultrafiltration, nanofiltration, and reverse osmosis [34–38], biodegradation by microorganisms [25], and chemical processes of base-catalyzed hydrolysis, ultraviolet (UV) radiation, ozonation, combined UV radiation/ozonation, catalytic ozonation, and combined UV/catalytic ozonation [25, 26, 39, 40]. Among the above said methods, adsorption can effectively remove the PAEs from the solution with PAEs concentrated on the solid adsorbents. The adsorption has been also applied to other emerging contaminants [41, 42]. The exhausted adsorbents are regenerated for reuse. The treatment of waste regeneration solution containing high-concentration PAEs is then followed. It is usually carried out by the destruction processes such as biological and chemical treatments noted above. Concerning the improvement of adsorption rate, proper surface area, and easy recovery of adsorbent, adsorption using novel magnetic micro-nano size magnetic adsorbents has been developed and employed for the removal of inorganic pollutants [43–46]. This study applied the micro size magnetic polyvinyl alcohol (M-PVAL) for the adsorption removal of organic DMP.

## 2. Experimental 

### 2.1. Chemicals

The main chemicals used to synthesize the micro size adsorbents of magnetic polyvinyl alcohol M-PVAL include the following: iron(III) chloride-6-hydrate (FeCl_3_·6H_2_O, 99%), iron(II) tetrahydrate (FeCl_2_·4H_2_O, 99%), and ammonium hydroxide (NH_3_, 25%) from Merck KGaA (Darmstadt, Germany), oleic acid (CH(CH_2_)_7_COOH, extra pure reagent) and polyvinyl alcohol ((CHCH_2_OH)_*n*_, 99+%) from Nacalai Tesque, Inc. (Kyoto, Japan), vinyl acetate (CH_3_COOCHCH_2_, 99+%) and divinyl benzene (C_10_H_10_, 80%) from Sigma-Aldrich, Inc. (Steinheim, Germany), benzoyl peroxide (C_6_H_5_CO, 99%, Jia Hwa Chemical, Co., Ltd., Taipei, Taiwan), and methylene blue (Loba Chemie Pvt. Ltd., Mumbai, India). The emerging contaminant of dimethyl phthalate DMP (C_10_H_10_O_4_, 99.5%) was supplied by Hayashi Pure Chemical Industries Ltd. (Osaka, Japan).

### 2.2. Methods and Analyses

#### 2.2.1. Preparation of Micro Size M-PVAL

Magnetic Fe_3_O_4_ (M) was prepared by chemical coprecipitation method. Ferrous chloride and ferric chloride were firstly dissolved using distilled water at 85°C in nitrogen environment. It followed the addition of aqueous ammonia to form the magnetic suspension of precipitated Fe_3_O_4_. Oleic acid acting as a dispersion agent was immediately and slowly fed into the suspension liquid until the appearance of clear supernatant liquid. This thus yielded the magnetite M. It was then synthesized to form magnetic polyvinyl acetate (M-PVAC) by suspension polymerization. For performing this, PVAL was dissolved via distilled water at 60°C in nitrogen environment to provide background solution for polymerization. After addition of magnetite M, VAC, and divinyl benzene, the suspension polymerization proceeded at 70°C for about 6 h and then cooled to 25°C, forming M-PVAC. Washing with deionized water, its surface was modified by alcoholysis to produce a polymer adsorbent of magnetic polyvinyl alcohol M-PVAL. In alcoholysis, M-PVAC was suspended in methanol solution for 6 h to obtain M-PVAL. Detailed description of the above procedures can be found in Tseng et al. [45].

#### 2.2.2. Isothermal Adsorption

DMP solutions with various concentrations were prepared. 0.1 g M-PVAL adsorbent was added into each 50 mL DMP solution filled in 125 mL flask. The initial pH value (pH_0_) of solution was adjusted to desired value using HCl or NaOH. The adsorptions with various initial DMP concentrations were conducted in a constant-bath shaker. A blank solution without M-PVAL adsorbent was tested along each batch of adsorptions.

After the adsorption of 8 h, a magnet was attached beneath the flask to adhere the M-PVAL adsorbent. The pH value of solution was measured. The magnetic separated solution was withdrawn using syringe and filtered with 0.22 *μ*m filter. 2 mL filtrate was collected for the measurement of concentration.

#### 2.2.3. Analyses

The magnetizations of Fe_3_O_4_, M-PVAC, and M-PVAL were measured by superconducting quantum interference device (SQUID) (model MPMS7, Quantum Design Inc., San Diego, CA). The Brunauer-Emmett-Teller (BET) surface area and particle size distribution of samples were determined by Perkin Elmer's Micromeritics ASAP 2000 (Perkin Elmer, Norcross, GA) and laser particle size analyzer (model LS 23 Fluid Module, Beckman Coulter Inc., Fullerton, CA), respectively. The surface structure and functional group of solids were analyzed using scanning electron microscope (SEM) (model JEOL-5610, JEOL Ltd., Tokyo, Japan) and Fourier-transform infrared spectrometer (FTIR) (model FTS-40, BIO-RAD Inc., Hercules, CA), respectively. A zeta potential meter (model Nano-Z, Malvern Inc., Worcestershire, UK) was employed to identify the zeta potentials of samples. The concentration of DMP was measured using UV-spectrophotometer (model UV mini-1240, Shimazu, Kyoto, Japan).

## 3. Results and Discussion 

### 3.1. Characteristics of Micro Size M-PVAL

**Figure 1:**
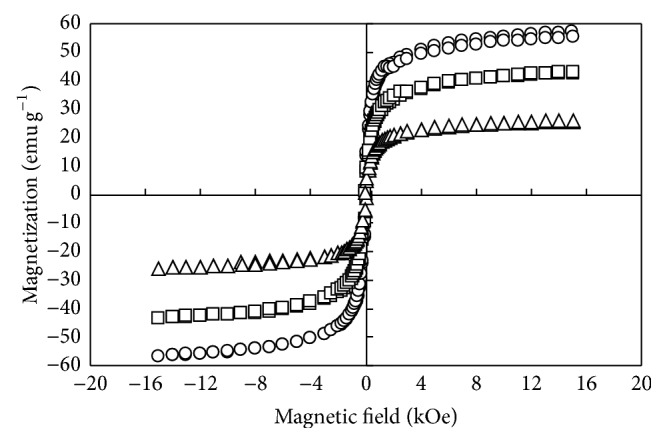
Magnetization curves of various magnetic particles. ○, □, and △: Fe_3_O_4_, PVAL: [CH_2_CH(OH)]_*n*_, and PVAC: [CH_2_CH(OCOCH_3_)]_*n*_.

Figure 1 illustrates the magnetization versus magnetic field of Fe_3_O_4_, M-PVAC, and M-PVAL. All three particles exhibit magnetization at the presence of magnetic field while they possess no magnetization in the absence of externally applied magnetic field, showing the unique characteristics of superparamagnetism. This property offers the advantages for using the fresh solid adsorbent to adsorb the solute species in the absence of magnetic field and for removing the exhausted adsorbent from solution by magnetic separation for subsequent regeneration. The saturation magnetizations of Fe_3_O_4_, M-PVAC, and M-PVAL are 57.2, 26.0, and 43.2 emu g^−1^, respectively. The saturation magnetizations of Fe_3_O_4_ and M-PVAC are close to those of Chang et al. [43] of 56.45 emu g^−1^ and of Tseng et al. [45] of 25 emu g^−1^, respectively. The reduction of saturation magnetizations of M-PVAC and M-PVAL compared to that of Fe_3_O_4_ with relative magnitudes of 45.45% and 75.52%, respectively, is due to the coating of polymer matter onto Fe_3_O_4_. The functional group of –OCOCH_3_ of M-PVAC is modified to –OH of M-PVAL, resulting in less organics contained in M-PVAL. Thus, for the same amount of magnetic adsorbent, the content of Fe_3_O_4_ in M-PVAL is higher than that in M-PVAC, possessing a comparatively higher saturation magnetization.

**Figure 2:**
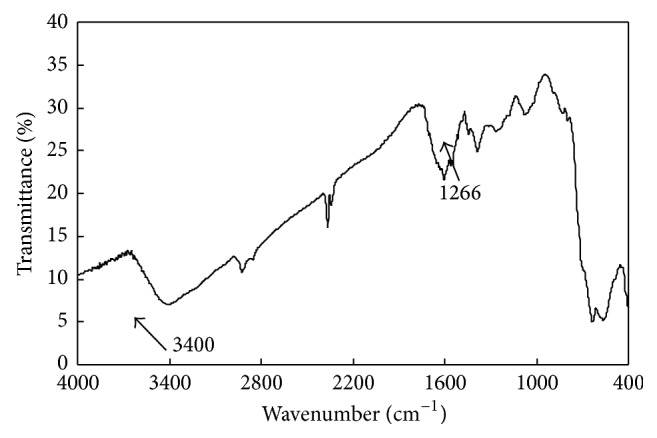
FTIR diagram of M-PVAL.

The success of synthesis of M-PVAL with functional group of –OH can be further justified by the peak of adsorption (inverse of transmittance) at 3400 cm^−1^in FTIR diagram presented in Figure 2. The peak at 1266 cm^−1^ also reveals the binding of C–O. The same characteristics of nonmagnetic polyvinyl alcohol were also reported by Kaczmarek et al. [47] and Majumdar and Adhikari [48].

**Figure 3:**
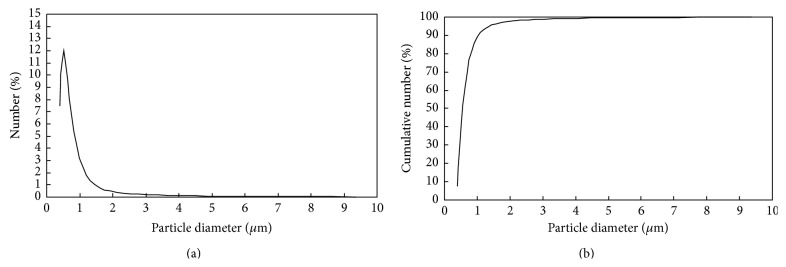
The relationship between (a) differential number % of M-PVAL and *d*_*P*_ and (b) cumulative number % and *d*_*P*_.

Figure 3 depicts the particle size distribution of M-PVAL in terms of number. The number mean diameter (*d*_PNA_) and number median diameter (*d*_PNM_) are 0.75 and 0.589 *μ*m, respectively. Most of the M-PVAL particles have size less than 1 *μ*m.

The major physical characteristics of M-PVAL are summarized in Table 1. The particle porosity *ε*_*P*_ is 0.03, indicating the micro size M-PVAL is essentially nonporous. This ensures that the pore diffusion is negligible in adsorption process.

### 3.2. Isothermal Adsorption of DMP

**Figure 4:**
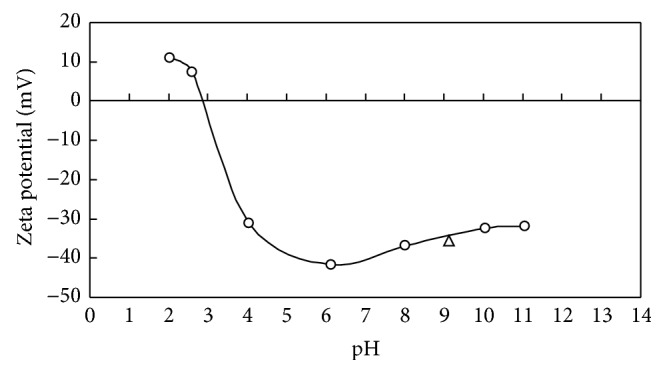
Zeta potentials of M-PVAL at various pH values. ○ and △: pH values of aqueous solutions containing M-PVAL are (1) controlled with addition of HCl or NaOH and (2) not controlled.

Langmuir, Freundlich, and D-R isotherms were tested to examine the adsorptions of DMP on M-PVAL for three cases, namely, Cases 1 and 3 with initial pH_0_ adjusted at 5 and 7 and Case 2 without adjustment of pH_0_ with pH value in 6.04–6.64, respectively. At equilibrium, the pH values for the three cases with pH_0_ = 5, 6.04–6.64, and 7 increase to about 7.36–7.87, 6.9–8.05, and 7.42–8.39, respectively. This is consistent with the adsorption of slightly acidic DMP by base M-PVAL, which exhibits pH of 9.1 and zeta potential of −35.6 mv when dispersed in deionized (DI) water as illustrated in Figure 4. The values of isotherm parameters of corresponding isotherms are listed in Table 2.

**Figure 5:**
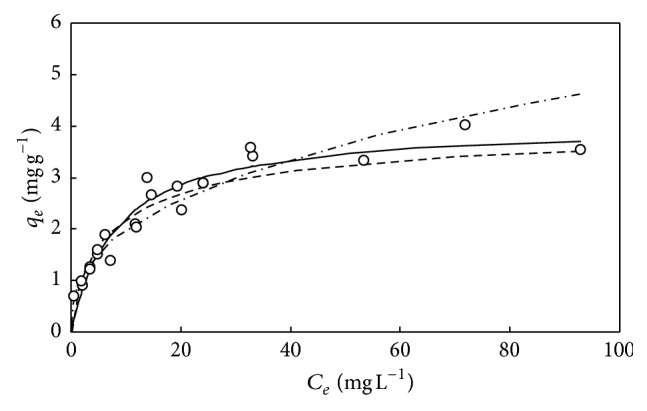
The simulations of Langmuir isotherm (—), Freundlich isotherm (- · -), and D-R isotherm (—) for adsorption of DMP on M-PVAL with pH_0_ = 5. Final pH = 7.36–7.87. ○: experimental data.

**Figure 6:**
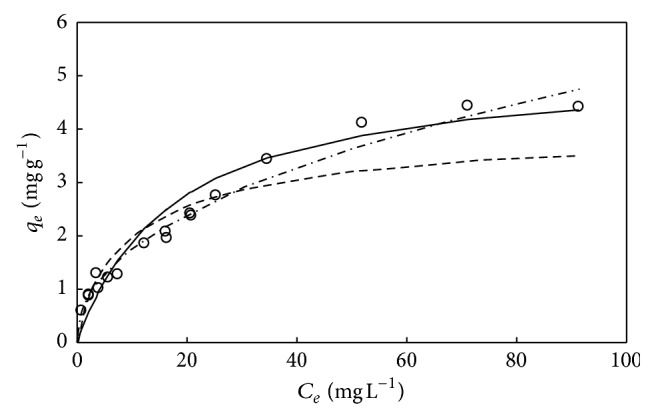
The simulations of Langmuir isotherm (—), Freundlich isotherm (- · -), and D-R isotherm (—) for adsorption of DMP on M-PVAL. pH_0 _= 6.04–6.64. Final pH = 6.9–8.05. ○: experimental data.

**Figure 7:**
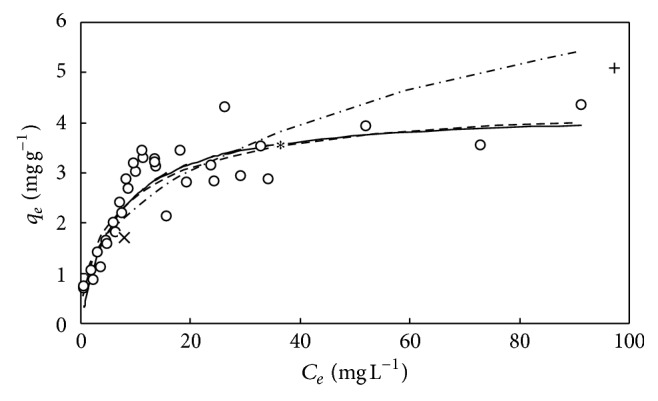
The simulations of Langmuir isotherm (—), Freundlich isotherm (- · -), and D-R isotherm (—) for adsorption of DMP on M-PVAL with pH_0_ = 7. Final pH = 7.42–8.39. ○: experimental data. ×, *∗*, and +: *q*_*e*_ versus *C*_*e*_ with *C*_0_ = 9.55, 40.0, and 102.4 mg L^−1^.

**Figure 8:**
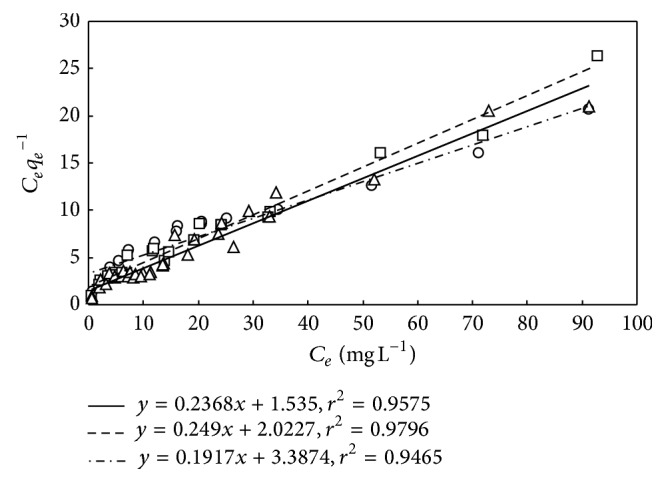
Adsorption of DMP on M-PVAL employing Langmuir isotherm. Cases with pH_0_ = 5, 6.04–6.64, and 7 are expressed as □ (–): *y* = 0.249*x* + 2.20227, ○ (- · -): *y* = 0.1917*x* + 3.3874, and △ (—): *y* = 0.2368*x* + 1.535.

The Langmuir isotherm deduces the values of unimolecular layer *q*_L_ of 4.01, 5.21, and 4.22 g kg^−1^ for Cases 1, 2, and 3, respectively, indicating minor effect of adjustment of pH_0_ on the saturation capacity *q*_L_ with Case 2 without adjustment of pH_0_ yielding higher value. The adsorption equilibrium constants *K*_L_ exhibit difference with Cases 1 and 2 with adjustment of pH_0_ giving higher values. The cause might be due to the effects of adjustment addition of HCl or NaOH on the adsorption. The balance of decrease of *q*_L_ while increase of *K*_L_ by the adjustment of pH_0_ results in the close adsorption behaviors for the three cases as depicted in Figures 5, 6, and 7. The *r*-squares of model fittings as shown in Figure 8 are greater than 0.94, indicating good agreement. The results thus suggest performing the adsorption without adjustment of pH_0_.

**Figure 9:**
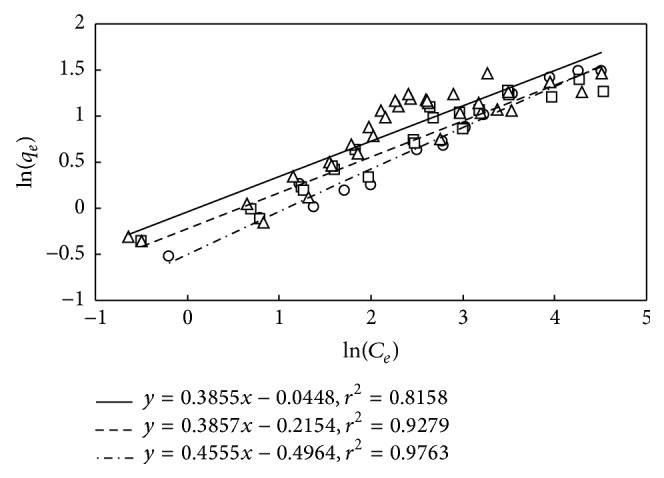
Adsorption of DMP on M-PVAL employing Freundlich isotherm. Cases with pH_0_ = 5, 6.04–6.64, and 7 are expressed as □ (–): *y* = 0.3857*x* − 0.2145, ○ (- · -): *y* = 0.4555*x* − 0.4964, and △ (—): *y* = 0.3855*x* − 0.0448.

The heterogeneity factors *n*_F_ of Freundlich isotherm obtained for the three cases are 2.59, 2.19, and 2.59, respectively. All these values are greater than 1, revealing that the adsorption of the noted DMP/M-PVAL systems is favorable. The *k*_F_ values are about 0.6–0.95 (mg g^−1^)(g m^−3^)^−1/*n*_F_^. The fittings of Freundlich isotherm as illustrated in Figure 9 are fairly satisfactory with *r*-square higher than 0.81, which, however, is not as good as those of Langmuir isotherm. The good fitting of Langmuir isotherm for the adsorbent M-PVAL may be further justified by noting that the M-PVAL is tiny with number average particle size of 0.75 *μ*m which exhibits fast adsorption rate with low diffusion resistance thus in favor of the formation of thin monolayer.

**Figure 10:**
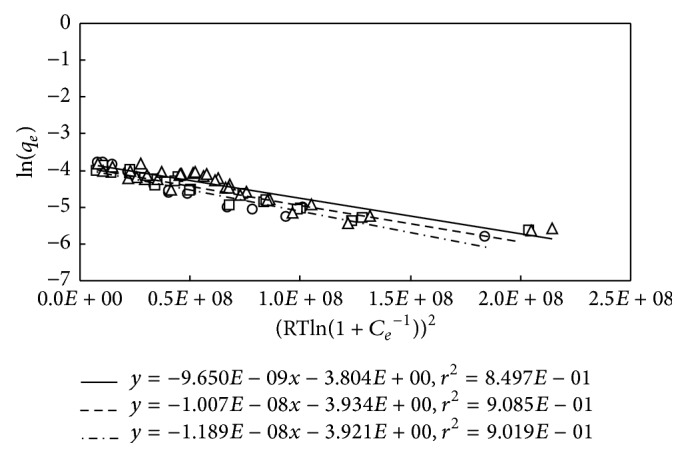
Adsorption of DMP on M-PVAL employing Dubinin-Radushkevich isotherm. Cases with pH_0_ = 5, 6.04–6.64, and 7 are expressed as □ (–): *y* = −1.007*E* − 08*x* − 3.934, ○ (- · -): *y* = −1.189*E* − 08*x* − 3.921, and △ (—): *y* = −9.950*E* − 09*x* − 3.804.

Applying D-R isotherm for the three cases gives the saturation adsorption capacity *q*_*m*D_ of 3.8, 3.84, and 4.32 g kg^−1^, respectively. The values are comparable to those of *q*_L_ of 4.01, 5.21, and 4.22 g kg^−1^ adopting Langmuir isotherm, further supporting the validity of Langmuir isotherm. The adsorption activation energies per mole of D-R isotherm *E*_D_ are 7.04, 6.48, and 7.19 kJ mol^−1^, respectively. The *r*-squares of fittings of D-R isotherm presented in Figure 10 are higher than 0.85, showing fair agreement. The obtained values of *E*_D_ of 6.48–7.19 kJ mol^−1^ are lower than those of 8–16 kJ mol^−1^ reported by Özcan et al. for the adsorption of ion-exchange form [49]. The low values of *E*_D_ thus support that the adsorption process of DMP/M-PVAL in this study proceeds naturally.

The above results indicate that the equilibrium of DMP/M-PVAL exhibits saturation value. Further, among the three isotherms examined, the Langmuir isotherm shows the best agreement and thus is more appropriate to describe the adsorption equilibrium of DMP/M-PVAL system.

## 4. Conclusions 

Some major conclusions may be drawn from the adsorption removal of DMP using superparamagnetic micro size adsorbent of M-PVAL examined in this study as follows:

1. Fe_3_O_4_, M-PVAC, and M-PVAL solid particles prepared possess superparamagnetism with saturation magnetizations of 57.2, 26.0, and 43.2 emu g^−1^, respectively. The magnetic particle with more organic adsorbent content has lower magnetization.
2. The M-PVAL can adsorb the DMP under the condition without externally applied magnetic field, while the exhausted saturated M-PVAL can be magnetically separated from the treated liquid for the regeneration by applying externally magnetic field.
3. Langmuir, Freundlich, and Dubinin-Radushkevich (D-R) isotherms were tested to describe the equilibrium of DMP/M-PVAL for three cases: (1) adjusting initial pH (pH_0_) at 5, (2) no adjustment of pH with pH_0_ = 6.04–6.64, and (3) adjustment of pH_0_ at 7, indicating that Langmuir isotherm offers the best fitting with good agreement.
4. The unimolecular layer *q*_L_ of Langmuir isotherm is 4.01, 5.21, and 4.22 mg g^−1^, respectively, for the three cases examined, suggesting no adjustment of pH_0_ for practical application.
5. For the three cases investigated, the adoption of Freundlich isotherm gives heterogeneity factor *n*_F_ of 2.59, 2.19, and 2.59, respectively, indicating that the said adsorption is favorable with *n*_F_ greater than 1.
6. The adsorption activation energies per mole *E*_D_ of D-R isotherm deduced are, respectively, of low values of 7.04, 6.48, and 7.19 kJ mol^−1^ for the three cases studied, supporting the natural occurrence of the adsorption of DMP/M-PVAL system.

## Figures and Tables

**Table 1:** Some major physical characteristics of M-PVAL.

		Adsorbent: M-PVAL
Density analysis	Bulk density, *ρ*_*P*_ (g cm^−3^)	2.40
	True density, *ρ*_*S*_ (g cm^−3^)	2.49
	Particle porosity, *ε*_*P*_^a^	0.03

Surface area^b,c^	Surface area by BET analysis (*A*_*B*_) (m^2^ g^−1^)	73.1
	Constant of BET equation (*K*_*B*_) (—)	63

Particle diameter^d^ (*μ*m)	Average particle diameter by number	0.75

^a^Calculated using 1 − (*ρ*_*P*_/*ρ*_*S*_).

^b^BET surface area was analyzed using ASAP2000, Micromeritics.

^c^Surface area estimated using Langmuir equation (*A*_L_) is about

102.42 m^2^ g^−1^.

^d^Analyzed using LS 23 Fluid Module, Beckman Coulter.

**Table 2:** Values of isotherm parameters and correlation coefficients (*r* ^2^)^a^ with different initial pH values.

pH_0_^b^	Langmuir isotherm			Freundlich isotherm			Dubinin-Radushkevich isotherm			
	*q*_L_ (mg g^−1^)	*K*_L_ (m^3^ g^−1^)	*r*_L_^2^	*k*_F_ ((mg g^−1^)(g m^−3^)^−1/*n*F^)	*n*_F_	*r*_F_^2^	*B*_D_ (mol^2^ J^−2^)	*E*_D_ (kJ mol^−1^)	*q*_*m*D_ (mg g^−1^)	*r*_D_^2^
5^c^	4.01	0.12	0.97	0.80	2.59	0.92	1.00 × 10^−8^	7.04	3.8	0.91
6.04–6.64^c^	5.21	0.056	0.94	0.60	2.19	0.97	1.19 × 10^−8^	6.48	3.84	0.90
7^c^	4.22	0.15	0.95	0.95	2.59	0.81	0.96 × 10^−8^	7.19	4.32	0.85

^a^ *r*_L_^2^, *r*_F_^2^, and *r*_D_^2^ are correlation coefficients by means of fitting the experimental data to Langmuir, Freundlich, and Dubinin-Radushkevich isotherms, respectively.

^b^The ranges of the equilibrium concentrations of the experiments performed are 0.61–92.9, 0.82–91.1, and 0.52–91.3 mg L^−1^ for pH_0_ of 5, 6.04–6.64, and 7, respectively.

^c^The final pH values of solutions at the end of experiments were about 7.36–7.87, 6.9–8.05, and 7.42–8.39 for pH_0_ = 5, 6.04–6.64, and 7, respectively.

## Abbreviations

*A*_*B*_: Surface area by BET analysis (m^2^ g^−1^)
*A*_L_: Surface area estimated using Langmuir eq. (m^2^ g^−1^)
*B*_D_: D-R isotherm constant (mol^2^ J^−2^)
BBP: Benzyl butyl phthalate
BET: Brunauer-Emmett-Teller
*C*: Adsorbate concentration in the liquid phase (g m^−3^ or mmol L^−1^)
*C*_*e*_: Adsorbate concentration in the liquid phase at equilibrium (g m^−3^ or mmol L^−1^)
D-R: Dubinin-Radushkevich
D-R isotherm: *q*_*e*_ = *q*_*m*D_exp⁡(−*B*_D_ *E*_*P*_^2^)
DBP: Di-n-butyl phthalate
DCHP: Di-cyclohexyl phthalate
DEHP: Di-(2-ethyl hexyl) phthalate
DEP: Di-ethyl phthalate
DHP: Di-hexyl phthalate
DIDP: Di-iso-decyl phthalate
DINP: Di-iso-nonyl phthalate
DMP: Dimethyl phthalate
DNOP: Di-n-octyl phthalate
*d*_*P*_: Particle diameter
*d*_PNA_: Number mean diameter
*d*_PNM_: Number median diameter
*E*_D_: Adsorption activation energy per mole of D-R isotherm (kJ mol^−1^), = 1/(2*B*_D_)^0.5^
*E*_*P*_: Polanyi potential (J mol^−1^), = *RT*ln⁡(1 + *C*_*e*_^−1^)
ECs: Emerging contaminants
FTIR: Fourier-transform infrared spectrometer
Freundlich isotherm: *q*_*e*_ = *k*_F_ *C*_*e*_^1/*n*_F_^
*K*_*B*_: Constant of BET equation (—)
*K*_L_: Equilibrium constant of Langmuir isotherm (m^3^ g^−1^)
*k*_F_: Constant of Freundlich isotherm ((mg g^−1^)(g m^−3^)^−1/*n*_F_^)
Langmuir isotherm: *q*_*e*_ = *q*_L_ *K*_L_ *C*_*e*_/(1 + *K*_L_ *C*_*e*_)
M: Magnetic Fe_3_O_4_
M-PVAC: Magnetic polyvinyl acetate
M-PVAL: Magnetic polyvinyl alcohol
*n*_F_: Heterogeneity factor of Freundlich isotherm (—)
PAEs: Phthalate acid esters or phthalate esters
PE: Polyethylene
PP: Polypropylene
PS: Polystyrene
PVC: Polyvinyl chloride
pH_0_: Initial pH value
*q*: Adsorbate concentration in solid phase (mg g^−1^ or g kg^−1^ or mmol g^−1^)
*q*_*e*_: Adsorbate concentration in solid phase at equilibrium (mg g^−1^ or g kg^−1^ or mmol g^−1^)
*q*_L_: Unimolecular layer of Langmuir isotherm (mg g^−1^ or g kg^−1^)
*q*_*m*D_: D-R isotherm constant denoting saturation adsorption capacity (mg g^−1^ or mmol g^−1^)
*R*: Universal gas constant (8.314 J mol^−1^ K^−1^)
*r*_L_^2^, *r*_F_^2^, and *r*_D_^2^: Correlation coefficients by means of fitting the experimental data to Langmuir, Freundlich, and D-R isotherms, respectively
SEM: Scanning electron microscope
SQUID: Superconducting quantum interference device
*T*: Absolute temperature (K)
UV: Ultraviolet.

### Greek

*ε*_*P*_: Adsorbent porosity
*ρ*_*P*_: Apparent particle density (kg m^−3^)
*ρ*_*s*_: True particle density (kg m^−3^)
*ρ*_*w*_: Density of water (kg m^−3^).
